# The influence of intentions on dream content

**DOI:** 10.1093/sleepadvances/zpae088

**Published:** 2024-11-28

**Authors:** Julia Fechner, Maren Born, Massimiliano Mancini, Zeynep Akata, Philipp Haag, Susanne Diekelmann, Jan Born

**Affiliations:** Institute of Medical Psychology and Behavioral Neurobiology, University of Tübingen, Tübingen, Germany; Institute of Medical Psychology and Behavioral Neurobiology, University of Tübingen, Tübingen, Germany; Department of Information Engineering and Computer Science, Multimedia and Human Understanding Group, University of Trento, Trento, Italy; Chair of Interpretable and Reliable Machine Learning, Technical University of Munich, Munich, Germany; Institute for Explainable Machine Learning, Helmholtz Munich, Munich, Germany; Institute of Medical Psychology and Behavioral Neurobiology, University of Tübingen, Tübingen, Germany; Institute of Medical Psychology and Behavioral Neurobiology, University of Tübingen, Tübingen, Germany; Department of Psychiatry and Psychotherapy, University Hospital Tübingen, Tübingen 72070, Germany; Institute of Medical Psychology and Behavioral Neurobiology, University of Tübingen, Tübingen, Germany; Werner Reichert Center for Integrative Neuroscience, University of Tübingen, Tübingen, Germany; German Center for Mental Health (DZPG), Tübingen, Germany; German Center for Diabetes Research (DZD), Institute for Diabetes Research and Metabolic Diseases of the Helmholtz Center Munich at the University Tübingen (IDM), Tübingen, Germany

**Keywords:** dreams, intentions, prospective memory, natural language processing

## Abstract

**Study Objectives:**

The “Zeigarnik effect” refers to the phenomenon where future intentions are remembered effectively only as long as they are not executed. This study investigates whether these intentions, which remain active during sleep, influence dream content.

**Methods:**

After an adaptation night, each of the 19 participants (10 women and 9 men) received three different task plans in the evening before the experimental night, each describing how to perform specific tasks. One of the task plans (completed) was then to be executed before the sleep period, another task (uncompleted) was told to be executed in the next morning, and on the third task (interrupted) participants were interrupted during the enactment before sleep and told to resume it the next morning. Polysomnography and multiple awakenings were conducted, resulting in 86 dream reports, 36 in NREM stage 2, and 50 in rapid eye movement sleep. After a traditional rating-based analysis of dream reports yielded inconsistent results, we analyzed the reports using a transformer-based assessment of dream incorporation, which quantified the semantic similarity between the dreams and pre-sleep tasks.

**Results:**

The number of dreams showing above-criterion similarity to the respective task was significantly lower for the completed than the uncompleted or interrupted tasks (*p* < .05, χ^2^ test). This pattern was confirmed through a forced choice approach, where—based on the similarity of single sentences of the dream reports—each dream report was allocated to one of the three task plans (*p* < 0.01, one-tailed χ^2^ test).

**Conclusions:**

Active intentions increase the likelihood of dream content being semantically similar to these intentions.

**Statement of Significance**

We used an objective AI-based large language model analysis to analyze the semantic similarity of dream reports to task plans participants had learned before sleep, and that was either executed before sleep or remained uncompleted and, thus, “active” during the subsequent sleep period. We find that the content of the reported dreams was more similar to the task plans that remained uncompleted than to task plans completed before sleep. Whereas dreams are well known to incorporate past experiences, these findings provide first-time experimental evidence that dreams also incorporate anticipated experiences, i.e. prospective memories for future plans.

Dreams occurring during sleep are known to incorporate memories of past experiences that occurred in a state of wakefulness, with the precise relationship between the waking experience and dream content representing one central question in this research field [1–4]. How are dreams constructed from waking experiences? Dream content appears to be particularly linked to new learning, although this idea is controversially discussed [2–4]. In fact, novel waking-life experiences have been shown to be incorporated into the content of non-rapid eye movement (NonREM) dreams, and this incorporation appeared to be specifically associated with the formation of new memories [5–7] suggesting that dreams reflect memory processing during sleep. Moreover, dream incorporation is enhanced when the task experience is highly engaging, e.g. through computer games [8, 9], virtual environments [10, 11], or stressful situations [12], i.e. all factors known to also enhance learning and the formation of new memories. In light of this link to memory formation, dream content has been proposed to be influenced by the neuronal replay of memory representations during sleep, as a core mechanism underlying memory consolidation [7–10, 13, 14]. Such influence of ongoing memory processing on dream content may extend—beyond the precise replay of cell ensemble firing patterns that occurred during learning—also to the reactivation of networks (semantically) connected to the replaying ensembles during sleep [15–19]. It is, however, important to note that although memory reprocessing can bias the dream content, the dream is rarely a mere replay of an episodic wake experience per se, but rather incorporates individual aspects connected to the waking experience [20].

Memories in the brain do not merely serve to represent the past but to predict the future. A first experimental demonstration of this future-oriented aspect of memory has been provided by Bluma Zeigarnik in 1927 [21], who showed that participants remembered tasks better that were interrupted and that remained to be completed in the future than tasks they had completed. The effect was explained such that a task that has already been started establishes a task-specific “tension” which improves accessibility of the relevant task memories, and which is only relieved upon completion of the task. The Zeigarnik effect has been confirmed and elaborated in numerous recent experiments on the nature of prospective memory for intentions and action plans (e.g. [22, 23]). Importantly, this research indicated that prospective memories for plans strongly benefit from periods of post-encoding sleep [24–28]. Specifically, this benefitting effect of sleep was significantly stronger for action plans that remained uncompleted before the experimental sleep period (due to an experimental interruption of task performance or an instructed delayed execution) than for plans that were completed before the sleep period [29].

Evidently, memory shares its distinct prospective orientation with dreams which have been considered “the brain’s offline efforts to distill projections of the future” [30]. This shared prospective focus might be the factor biasing dream content toward the acutely processed memories. Specifically, we propose that dreams incorporate more elements from task plans made during wakefulness, if the respective task is not yet completed and the intention to complete the plan is kept active across sleep. To test this, participants were given three different task plans before sleep. One plan was completed before the experimental sleep period (completed). Of the remaining two plans, one was never started by the participants before sleep, as they were informed it would need to be completed the next morning (uncompleted). The other plan was started but interrupted before sleep, with participants instructed to finish it the following morning (interrupted).

Crucially, we used an objective approach to analyze the similarity between the task plans and the dream reports resulting from multiple awakenings during the postacquisition sleep period. Recently, strong efforts have been made to objectively analyze dreams [31–37], with particularly impressive results from neural decoding approaches in which machine-learning models predict the reported dream contents based on measured brain activity [38]. Here, we deployed a large language model to match objectively a dream report with the respective task plans, with the results from this objective analysis confirming that the obtained dream reports were on average more similar to the uncompleted and interrupted task plans than to the completed task plan.

## Methods

### Participants

A total of 20 young, healthy participants aged between 19 and 29 years (M = 24.15 ± 3.08) took part in the study, including 11 female and 9 male participants. All participants were native German speaking, non-smokers, and reported not to suffer from any neurological or psychiatric conditions. They did not take any medications (except oral contraceptives), iron, or vitamin supplements. Individuals reporting difficulties to fall or stay asleep, nightmares, or other sleep disorders were also excluded. All participants reported to follow a regular sleep/wake schedule with >6 hours of sleep per night and no shift work, night duties, or long-distance flights with jet lag in the 6 weeks prior to the experiment. Only participants were included who assessed themselves as individuals “dreaming on occasion” and normally having at least “fairly good recall” of their dreams.

Participants spent one adaptation night in the sleep lab before the experimental night, in order to familiarize them to sleeping with EEG electrodes. Data from one participant were excluded from the analysis because of too poor sleep quality, reducing the sample to a total of *N* = 19 participants. Before the start of each experimental night, the current well-being and health of the participants were assessed, including whether they had experienced any unusual kind of stress, and had refrained from napping, alcohol, and caffeine on that day before the experimental night. Participants gave written informed consent and were paid for participation. The study was approved by the local ethics committee of the University of Tübingen.

### Design and procedures

The study followed a within-subject design, examining the correspondence of dream reports obtained in a single experimental night to each of three different task plans with different execution status (completed, uncompleted, and interrupted). The task plans were entitled “Setting the table,” “Tidying the desk,” and “Getting ready to leave.” The first two scripts were derived from another study [39], while the third one was newly developed for the purpose of this study. Each task plan was assigned to one of the three execution statuses. Participants learned the scripts for all task plans in the evening and performed them either afterward (for the completed and interrupted conditions) or in the next morning (uncompleted). The assignment of task plans to the execution status conditions was balanced across conditions (completed – C, interrupted – I, uncompleted – U) distributing as follows: “Setting the table” (C = 7; I = 7; U = 6), “Tidying the desk” (C = 6; I = 6; U = 8), “Getting ready to leave” (I = 7; C = 7; U = 6). Also the order of execution status conditions for the tasks to be performed in the evening was balanced across participants such that the “completed-interrupted” order occurred nine times and the “interrupted-completed” order occurred 11 times. During the nocturnal sleep period, dream reports were gathered during awakenings from either rapid eye movement (REM) sleep or NREM stage 2. The participants were informed that the experiment investigated the effect of sleep on memory for specific task plans and that awakenings for dream reporting would occur.

**Figure 1.**
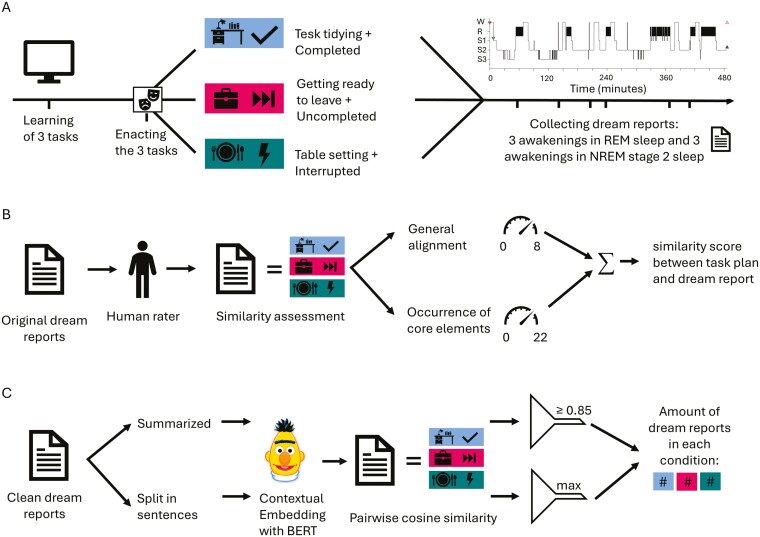
Experimental procedure and analysis. (A) Experimental procedure for an example participant who completed the task plan “Tidying the desk” before sleep, and was told to execute the task plan “Getting ready to leave” the next morning. On the “Table setting” task plan he/she was interrupted when implementing it before sleep and asked to complete it the next morning. Right: sleep profile for subsequent nighttime, indicated 6 awakenings for oral dream report collection. (B) Analysis of dream reports based on a traditional rating-based analysis of dream reports, where two human raters were asked to rate the similarity of dream reports to the task plans, relying on a standardized scale by Schredl [40]. The similarity score, here, combined points given for the rated overall similarity between a dream report and a specific task plan (0–8 points), and points given for the rated similarity of the reports with core elements of the respective task plans (0–22 points). (C) Analysis of dream reports based on an AI large language model (GermanBERT): dream reports were transcribed into written texts and filler words and grammatical errors were removed. Reports were then either automatically summarized or split into sentences. Semantic similarities between task plan descriptions and dream reports were then determined through contextual embedding and calculating cosine similarities. Summarized dream reports passing the 0.85 criterion threshold and maximum values of dream reports split into sentences, respectively, were counted for each execution status condition.

The experimental procedure is illustrated in Figure 1A. All subjects reported to the laboratory at 8:00 pm. First, a questionnaire regarding participant data was completed to ensure all experimental inclusion criteria were met. Then, EEG electrodes were attached, and the participants completed the Stanford Sleepiness Scale (SSS) and a mood questionnaire, followed by the Regensburg Word Fluency Test (RWT) and a Vigilance Task (VT), both assessing executive cognitive functions.

After performance on these tests, the participants learned the action sequences for the three task plans as described in [41]. The plans were entitled “Desk tidying,” “Getting ready to leave,” and “Setting the table.” Each task plan comprised an action sequence of five subtasks (Supplementary Figure 1). For Desk tidying the subtasks were (1) opening a file, (2) filing documents, (3) sharpening a pencil, (4) sorting index cards, and (5) stacking articles. For Getting ready to leave, the subtasks were (1) shutting down the computer, (2) closing the window, (3) putting on a coat, (4) putting on a backpack, and (5) switching off the light; and for Setting the table, the subtasks were (1) spreading out the tablecloth, (2) distributing tableware, (3) polishing glasses, (4) folding napkins, and (5) lighting candles. The subtasks were presented sequentially on a computer screen, with the title of the task plan displayed above each subtask. Every subtask was shown for 6 seconds in a fixed order. Following this sequential presentation, the task title and all five subtasks were presented together for 30 seconds to allow participants to consolidate the information. This process was repeated three times for each task plan, with one complete cycle through the three plans constituting a learning trial.

After the initial learning phase, an immediate recall test was performed where each task title was presented, and the participant was asked to recall (verbally) the five subtasks of the plan in the correct order. It was ensured that the participants memorized the exact wording and order of the subtasks. If they did not achieve 100% correct recall on all three task plans during two consecutive recall tests, additional learning trials were conducted.

The second (and following) learning trials followed the same procedure as the first, but with each task plan being presented only once. On average, participants required 2.9 ± 0.9 learning trials (range 2–5) to meet the recall criterion. To enhance task involvement, the participants were informed that they would receive an additional 15 € for correctly recalling all three task plans (5 € per plan).

After learning, the participants were informed which two task plans were to be performed in the evening and which one in the morning. Plans were executed under observation and evaluation by the experimenter, with the participant not being allowed to ask any questions while performing the task plans.

The materials used to execute the plans were prepared beforehand. Once the participant had performed on the first plan in the evening he/she left the room so that the materials could be reset for the next task plan. For the interrupted condition, the experimenter interrupted the execution of the task plan after the participant had completed the first subtask. Under the pretext that an error had occurred, the participant was instructed to perform this task the next morning. After performing the two task plans, the participants went to bed.

For the experimental sleep interval, lights were turned off at the participant’s habitual bedtime. After about 3 hours of sleep or latest at 2:00 am, the first awakening occurred. It was ensured by visual inspection of the ongoing polysomnographic recordings, that before an awakening the sleep stage was stable for at least 10 minutes. Awakenings were done up to six times per night, from NREM stage 2 and REM sleep to cover both NonREM and REM sleep-associated dreams [42–45]. Subsequent awakenings always were carried out after the participant had regained sleep for at least 30 minutes. For awakening, lights were turned on and the participant was addressed by their name and asked to sit up and put on a headset for voice recording. A standard set of questions followed: (1) Tell me everything that was going through your mind before you were woken up. (2) Can you remember any details? (3) And further? (4) Was it a dream or a thought? and (5) Was it pleasant, unpleasant, or neutral? Question 3 was repeated unless the participant explicitly reported not having any further memory. Lights were turned off for the participant to return to sleep once no further details came into their mind. The participant was awakened the next morning at about his/her usual wake-up time. The SSS, mood questionnaire, RWT, and VT were performed a second time about 30 minutes after awakening.

### Polysomnographic recordings

Throughout the night, the EEG was recorded from six channels (F3, F4, C3, C4, P3, and P4) referenced to two electrodes attached to the mastoids (M1 and M2) using a BrainAmp DC amplifier (BrainProducts, Munich, Germany). The ground electrode was placed on the forehead. Impedances were always kept below 5 kOhm. Additionally, vertical and horizontal eye movements were measured (VEOG and HEOG) as well as an electromyogram (from two electrodes placed on the chin). Signals were band-pass filtered between 0.3–30 Hz (EEG and EOG signals) and 5–150 Hz (EMG signal), sampled at 500 Hz and stored for offline analyses. Visual scoring of 30-second polysomnographic records followed the criteria outlined by Rechtschaffen and Kales [46].

### Dream report analysis

Audio-recorded dreams were transcribed into written texts before analysis. To quantify the extent to which the task plans at the different execution states were incorporated into the dream reports, we analyzed the semantic similarity between dreams and respective task plans. These similarity analyses were performed in two different ways, first using a traditional approach based on subjective ratings and, second, based on an AI large language model.

For the rating-based approach, two colleagues, sleep experts with no special experience in the assessment of dreams, were asked to rate the dream reports according to a standardized scale derived from a previous report by Schredl [40]. The first part of the scale required to rate the general extent of alignment between the reported dream and the specific task plans between 0 and 8 (indicating no vs. high correspondence). The second part of the scale aimed at a similarity rating based on the occurrence of certain core elements characterizing the task plans. For this, for each task plan, 11 core elements, i.e. objects, and activities that characterized the plan, were selected. The rating required to assign, for each of these core elements, a score between 0 and 2 with 0 indicating that the element did not occur in a dream report, 1 indicating that the element occurred metaphorically or indirectly, and 2 indicating that the element was directly named in the dream report, with the sum of the 11 scores defining the similarity between the dream report and the respective task plans. Raters were blinded as to the execution state conditions and as to whether a dream was reported after a NonREM or REM sleep awakening.

For the second analysis (illustrated in Figure 1B), we used a large language model to objectively quantify the extent of dream incorporation. Dream incorporation was measured by the degree of semantic similarity between the task plans and the dream reports. Semantic representations of the task plans and dream reports were extracted using the transformer-based representational model Bidirectional Encoder Representations from Transformers (BERT) [47], a natural text embedding model capable of quantifying semantic textual similarity [48]_._ Specifically, we used the German version (GermanBERT) [49] which is pretrained on written German texts, i.e. German Wikipedia articles, German OpenLegalData, and German news articles. The parameters of the model were trained by (1) splitting the input texts, i.e. the dream reports and task plans, into tokens representing semantic units (words, subwords) of the input texts, (2) masking some tokens and feeding back the corrupted sentence (with masked tokens) as input into the model, and (3) asking the model to reconstruct the original tokens.

Since BERT was pretrained on written language, the transcripts of the dream reports were additionally edited to remove filler words and to correct grammatical errors. Similarly, the task plans were prepared for these analyses by transforming the description of subtasks in bullet points into full sentences. Then, each dream report was summarized automatically using a BERT model fine-tuned for text summarization (https://huggingface.co/mrm8488/bert2bert_shared-german-finetuned-summarization) before giving it as input to the GermanBERT. The GermanBERT encoder embeddings (vectors that take into account single text units and respective semantic relationships to other units) were used as a representation of the dream. While these embeddings are influenced by sentence length and parts of speech, this information is not explicitly encoded in the embeddings. We then encoded each of the five subtasks in each of the three task plans into embeddings following the same procedure (without prior summarization). Cosine similarity between the embeddings was calculated resulting in a similarity score for each dream report with each action of the task plans, for which then the maximum similarity score for each task is chosen. Cosine similarity measures how closely aligned two vectors are, regardless of their magnitude, by calculating the cosine of the angle between them. In our study, we used cosine similarity to quantify the semantic relatedness between dream reports and task descriptions, with scores ranging from −1 (opposite meaning) to 1 (identical meaning). Because similarity cosine values for the dream reports fell into a more limited range at the higher end of the scale [50], we thresholded cosine values using only values greater or equal to 0.85 to focus the analysis on dreams with high similarity scores. We then compared the number of dream reports with the above criterion similarity scores for a given task plan, between the execution status conditions completed, interrupted, and uncompleted.

In a further sentence-wise analysis, we split the written dream reports into single sentences. Based on GermanBERT, we then calculated the cosine similarity score for each sentence of a dream report and each subtask of a task plan. The resulting maximum similarity value for each dream report was then used to allocate the dream report to one of the three execution status conditions according to a forced choice procedure.

### Statistical analyses

Similarity scores for dream reports were generally analyzed using analyses of variance (ANOVA) including repeated measures factors representing the executive status conditions (completed, interrupted, and uncompleted) and the sleep stage (NREM stage 2, REM) prior to the obtained report, with subsequent post-hoc *t*-tests used to specify the significance of pairwise comparisons. Pearson’s correlation coefficients were used to assess interrater reliability in the analyses based on subjective ratings. Given that similarity scores for the dream reports with the above criterion similarity in the AI language model-based analyses were not uniformly distributed among execution status conditions and task plans, we focused on the statistical analysis of these scores on nonparametric testing using χ^2^tests. Prior to testing, the number of dream reports above the criterion in each execution status condition and for each task plan was additionally divided by the total number of dream reports obtained for each execution condition and task plan. The χ^2^ test was also used to detect deviations from equal distributions of dream reports collected in different sleep stages, conditions, and tasks. The level of significance was set to *p* = .05 for all statistical tests, and *p* = .01, in case of directed one-tailed testing of hypotheses.

## Results

### Dream report acquisition

A total of 117 awakenings were performed, equally distributed across REM sleep and NREM stage 2, i.e. 59 (50.4%) awakenings were performed in REM sleep and 58 (49.6%) awakenings in NREM stage 2 sleep (*p* = .926, χ^2^ test). A dream was recalled in 86 (73.5%) of the awakenings. Of these 86 dream reports, 51 (59.3%) were collected after REM sleep awakenings and 35 (40.7%) after NREM stage 2 sleep awakenings, resulting in a trend toward more dream reports collected after REM sleep than NREM stage 2 awakenings (*p* = 0.084, χ^2^ test). Since participants’ ability to recall dreams at each awakening differed, the number of dream reports was not evenly distributed among task plans and execution status conditions (Table 1). SSS sleepiness scores averaged (mean ± standard deviation) 3.1 ± 0.85 in the evening before sleep, and 2.5 ± 0.76 in the next morning. Performance scores on the RWT averaged 16.4 ± 4.81, in the evening, and 16.6 ± 4.32 in the next morning.

**Table 1.** Total number of dream reports for the task plans and execution status conditions, across all awakenings (top) and separately for awakenings from REM sleep and NREM stage 2 sleep

All reports	Completed	Uncompleted	Interrupted	∑
Setting the table	34	25	27	86
Tidying the desk	20	37	29	86
Getting ready to leave	32	24	30	86
∑	86	86	86	258
Reports in REM				
Setting the table	19	16	16	51
Tidying the desk	13	21	17	51
Getting ready to leave	19	14	18	51
∑	51	51	51	153
Reports in NREM stage 2				
Setting the table	15	9	11	35
Tidying the desk	7	16	12	35
Getting ready to leave	13	10	12	35
∑	35	35	35	105

### Dream incorporation assessed by subjective ratings

The two raters only moderately agreed on their ratings of the dream reports. Although significant, both correlations between their ratings of the general similarity between task plans and dream reports as well as correlations of their judgments based on the occurrence of core elements of the task plans (objects, activities) in the individual dreams were only of medium size (0.52 < *r* < 0.65, *p* < .01, Pearson’s correlation). ANOVA performed on ratings collapsed across both raters did not reveal any significant difference in similarity ratings between any of the execution status conditions or awakenings from REM or NREM stage 2 (all *p* > .67 for respective ANOVA factors). Values collapsed across general similarity ratings and core element-based ratings indicated for one of the rater's highest dream incorporation for the completed task plans (1.02 ± 0.20), medium for uncompleted plans (0.98 ± 0.20), and lowest for interrupted task plans (0.80 ± 0.17) whereas for the other rater, values were highest for the interrupted task plans (1.20 ± 0.22), medium for completed plans (0.78 ± 0.14) and lowest for the uncompleted tasks (0.70 ± 0.15). The overall insufficient agreement between our raters is consistent with a great body of findings in this field of dream content analysis [51, 52] and led us to switch to an AI-based approach.

### Assessment of dream incorporation by an AI-based large language model approach

**Figure 2.**
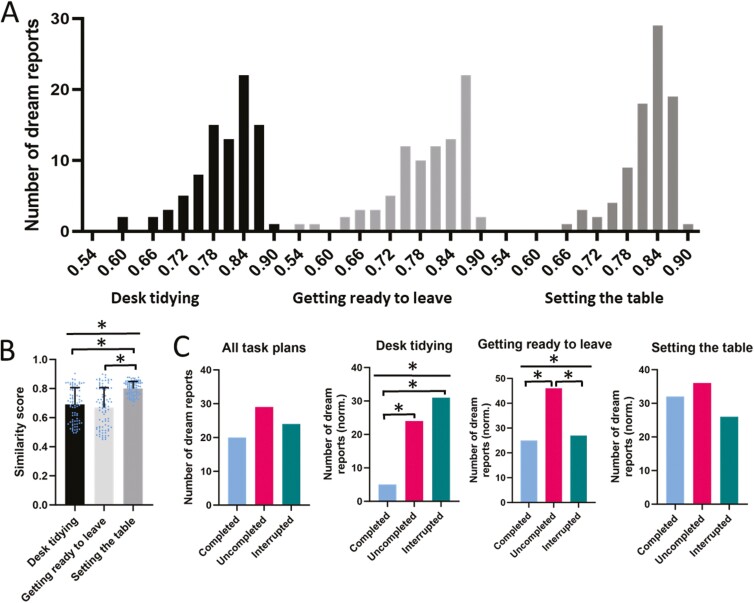
Whole-text similarity analysis of dream reports. (A) Frequency distribution of dream report similarity scores for each of the three task plans. (B) Mean ± SEM similarity scores for the task plans across execution status conditions. Note, that the generally enhanced number of reports with similar to the “Setting the table” task. Asterisks indicate *p* < .05, for ANOVA across the three task plans (straight line) and for (unpaired) two-sided *t*-tests between task plans (brackets). (C) Number of dream reports with similarity scores ≥0.85 across all task plans and separately for each task plan. To account for differences in the general similarity scores between the task plans, numbers for the separate plans were normalized by dividing the number of reports with ≥0.85 similarity with the total number for the respective plan. Asterisks indicate *p* < .05 two-tailed χ^2^ test, for the comparison across all three execution status conditions (straight line) and for pairwise comparisons between conditions (brackets).

Here, we determined the semantic similarity of dream reports by applying a large language model (GermanBERT) to transcripts of the reports (Figure 1B). Cosine similarity scores were calculated for the whole texts, indicating the similarity between a dream report and one of the three task plans. For all task plans, similarity scores ranged between 0.54 and 0.90, with maximum frequencies in the 0.84–0.90 range (Figure 2A), with this distribution indicating that a minimum similarity (of around 0.54) to each of the three task plans is basically reached by any of the dream reports. The direct comparison between task plans, on the other side, indicated that the task plan “Setting the table” yielded distinctly higher similarity scores than the two other task plans (*F*(1.040,88.43) = 116.9, *p* < .0001, for ANOVA main effect of task plan), with this effect being independent of the execution status condition the task plan was assigned to (*p* = .49 for respective interaction effect with execution status factor, Figure 2B). This finding points to an a priori difference in our task materials, with a higher likelihood for the “Setting the table” plan to be similar to a dream report than for the other two plans.

Given that the frequency distribution of similarity scores (Figure 2A) indicated that each dream report shows at least a minimum similarity to any of the three task plans (of around 0.54), we focused our analyses on only the dream reports exhibiting substantial similarity to one of the tasks, adopting a criterion similarity score of ≥0.85. Indeed, we assumed that higher similarity scores are associated with a higher probability that this similarity was related to specific features of one of the three task plans. Note, although the ≥0.85 criterion is arbitrary, virtually the same results were obtained with lower criteria, up to ≥0.75. Comparing each dream report with each of the three task plans, we revealed that out of all 86 dream reports, 29 reached a semantic similarity score ≥0.85 to the task plan assigned to the uncompleted execution status condition, 24 dream reports reached the ≥0.85 criterion for the interrupted tasks, and 20 for the completed task plans. Although descriptively this pattern concurred with our hypothesis of an increased incorporation into dream reports of contents from uncompleted and interrupted task plans in comparison with completed task plans, it did not reach significance (χ^2^(2,73) = 1.67, *p* = .4439, for the comparison between execution state conditions), which we hypothetically attributed to the fact that our task plans showed a priori differences in the likelihood of being highly similar to a dream report with the highest likelihood for the “Setting the table” task plan (Figure 2B). Indeed, analyzing separately similarity scores for the different task plans, we found that significantly more dream reports were semantically similar to the “Desk tidying” and “Getting ready to leave” task plans when they were uncompleted (Desk tidying—24, and Getting ready to leave—46) or interrupted (Desk tidying—31, and Getting ready to leave—27) compared to being completed (Desk tidying—5, Getting ready to leave—25; Desk tidying: *χ*^2^(2,60) = 18.10, *p* < .001; and Getting ready to leave: *χ*^2^(2,98) = 8.23, *p* < .05; for the comparison across all three execution state conditions, see Figure 2C for pairwise comparisons). On the other hand, for the “Setting the table” task plan no such pattern was obtained (χ^2^(2,99) = 2.24, *p* > .30). We found no comparable differences between the execution status conditions in separate analyses of dream reports obtained after REM sleep awakenings (all *p* > .36) or after NREM stage 2 sleep awakenings (all *p* > .33, χ^2^ test). Moreover, an exploratory control analysis of word counts for dream reports with the highest similarity to task plans revealed no significant differences between the execution status conditions (*p* = .5052, *F*(2,28) = 0.7053).

**Figure 3.**
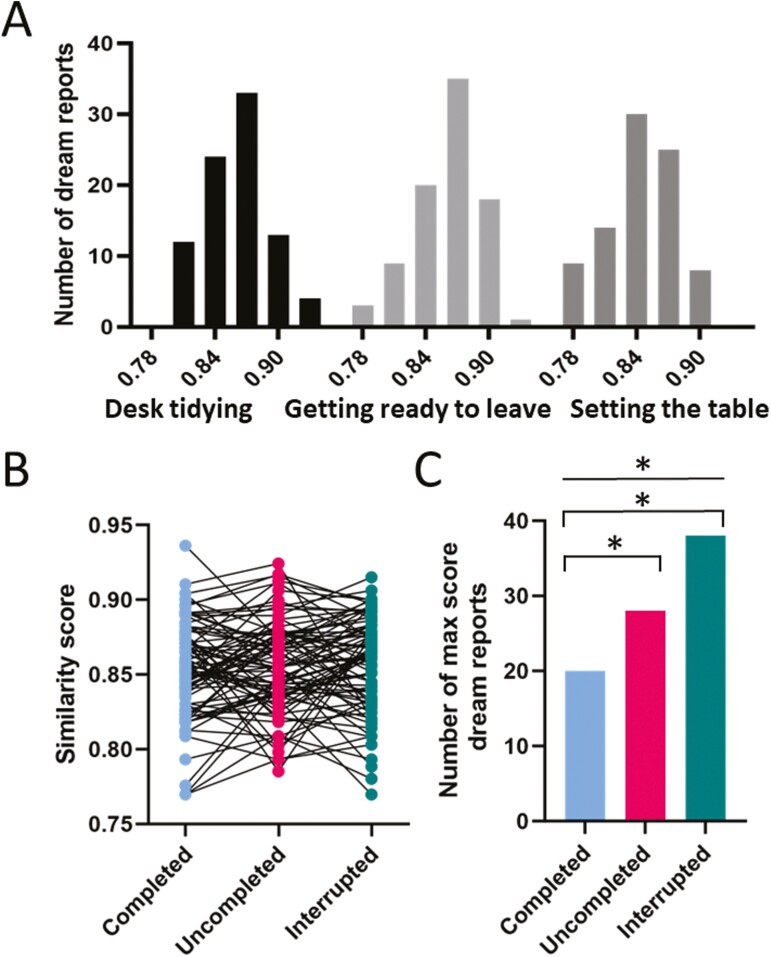
Single sentences-based similarity analysis of dream reports. (A) Frequency distribution of dream report similarity scores for each of the three task plans. (B) Similarity scores for each of the 86 dream reports for the tasks plans assigned to the completed, uncompleted, and interrupted condition in the respective session. Solid lines connect scores for the same dream report. (C) The number of dream reports with maximum similarity score, for each execution status condition. Each dream report was allocated to the execution status condition showing the maximum similarity score for this report. *p* < .05 one-tailed χ^2^ test, for the comparison across all three conditions (straight line) and for pairwise comparisons between execution status conditions (brackets).

To further validate our large language model-based approach, in a second analysis, we made use of a forced choice method where dream reports were allocated to one of the three execution status conditions after a sentence-wise comparison of dream reports and task plans (Figure 1B). This forced choice approach appeared to be also favorable against the backdrop that the similarity scores of an individual dream report for the three different task plans were generally rather close to each other, i.e. showed relatively low variability in comparisons with the high variability of similarity scores among the different dream reports (averaged across task plans; *F*(1,83) = 0.0014, *p* < .002, for a direct comparison between respective variances). This sentence-wise analysis revealed similarity scores ranging between 0.78 and 0.90 for each of the three task plans, with maximum frequencies around 0.86 (Figure 3A). Similarity scores again significantly differed for the three task plans (*F*(2, 249) = 8.7, *p* < .0005, for ANOVA main effect of task plan), with this effect being independent of the execution status condition the task plan was assigned to (*F*(4, 249) = 0.64, *p* = .63, for ANOVA task plan × execution status interaction). When we assigned each dream report to the execution status condition with the highest similarity score for this dream report, we found that the lowest number of dream reports, i.e. 20 reports, were assigned to the completed condition, the number of assigned reports was intermediate (28 reports) for Uncompleted task plans, and highest for interrupted task plans (38 dream reports (*χ*^2^(2,86) = 5.678, *p* < .01, one-tailed χ^2^ test, for the comparison across all three conditions, Figure 3B and C).

## Discussion

In this study, we explored whether intentions for future actions influence dream content. Employing an AI-based large language model analysis, we show that task plans that have not been completed before sleep and, hence, remain active during sleep, influence the content of a dream to a greater extent than tasks that are completed before sleep. Specifically, tasks whose execution was interrupted before sleep or whose execution was anticipated for the morning after sleep produced dream reports of greater semantic similarity to these tasks than task plans that were completed before sleep. Whereas firm evidence has been accumulated that dreams incorporate past experiences [10, 12, 33, 53], especially if they are emotional [54–56], our findings provide first-time experimental evidence that dreams also incorporate anticipated experiences, i.e. future plans.

In psychological terms, our findings relate to the well-known Zeigarnik effect [21] or the intention–superiority effect [39] which describes the phenomenon that a planned action is better retained in memory or in a heightened state of activation as long as the plan is not executed [57–59]. It is assumed that a “tension” sometimes carrying also an emotional tone [52], drives ongoing processing of memory representations connected to the plan, as long as it is not executed. This tension and associated processing of plan-related representations is not necessarily conscious, and we here provide evidence that it extends into sleep biasing the content of dream reports. This conceptual view is in line with multiple studies showing that sleep promotes problem solving on tasks that remained unsolved before sleep, probably due to a subliminal ongoing processing of the problem [2, 60, 61]. Indeed, the incorporation of experienced content into dreams has likewise been linked to an ongoing reprocessing of respective memory representations that not only supports the consolidation of respective memory but simultaneously, expresses itself in dream reports that are semantically biased toward the reprocessed memory contents [3, 5, 9, 10, 20].

Studies of prospective memories for plans and intentions have indicated a greater benefit for such memories of uncompleted plans from slow wave sleep (SWS) than REM sleep [26]. Hence, assuming a direct link between processes of memory consolidation and dreaming, one might expect that dream reports after awakening from SWS show a greater similarity to the uncompleted task plans than reports after REM sleep awakenings. The present data remain inconclusive in this regard for two reasons. First, rather than in SWS, we awakened the participants in NREM stage 2 sleep during which the reprocessing of the to-be-consolidated memory representations might be less intense than in SWS, though evidence for such difference is mixed [62–64]. Second, our analyses relied on a rather small number of dream reports. The data set, hence, did not provide sufficient statistical power for reliable analyses on subsets separating dream reports between awakenings from NREM stage 2 and REM sleep, considering the size of *f* = 0.48 (G*Power version 3.1.9.7) for the effect of the plan execution status on dream report similarity for our analysis across all (NREM stage 2 and REM sleep) dream reports.

Our finding of an incorporation of uncompleted task plans into dream reports appears to be especially noteworthy in that it derives from an objective large language model-based machine-learning approach, i.e. an approach which, except for a most recent study [65], has so far not been used for dream content analyses. This large language model-based approach overcomes the weakness of traditional analyses of dream reports based on subjective ratings which are notoriously unreliable suffering from modest inter-rater agreements [66, 67]. Here, using two independent raters to classify dream reports according to their similarity to the different task plans, we also found only rather low interrater reliability and, consequently no distinct differences in similarity between dream reports and task plans. Conceivably, we could have strengthened inter-rater reliability by a more intense prior training of our raters on “sham reports” including the discussion of discrepant scores between raters [67]. Perhaps, we could have also enhanced our ratings if—like in other studies [68, 69]—we had additionally asked our participants themselves to rate the similarity of their dream reports with the task plans. Exclusively relying on ratings by other persons, our analyses did not yield any conclusive results.

Nevertheless, although objective, our large language-model-based machine-learning approach bears several limitations, altogether calling for further confirmation of our main findings. First of all, we applied the large language model-based approach post-hoc, and only after the subjective ratings turned out to be unreliable. Related to this, our task plans were not particularly tailored for a large language model-based analysis of their semantic similarities. Basically, the task plans turned out to be too similar to each other, resulting in a large overlap between the task plans with respect to their similarity to the dream reports. While we adopted our task plans from a foregoing study [39] targeting the persistent activation of intentions in memory, task plans with greater semantic differences in the activities, objects, and contexts might have increased the differences in similarity between plans and dream reports. Interestingly, the “Setting the table” plan yielded significantly higher similarity scores than the two other task plans, suggesting that certain activities may be more prone to dream incorporation than others. Obviously, future studies adopting new task plans should rule out such a priori differences in task plans before experimental use. Such future studies should also overcome other limitations of our study arising, e.g. from a rather crude assessment of sleep lacking occipital recordings which may be particularly important for a precise sleep scoring assessment and therefore relevant in analyses of (visual) dreams.

Our large language model-based approach, using GermanBERT as an embedding extractor, revealed significant differences in dream report similarity that confirmed our a priori hypotheses, supporting the validity of this approach. However, statistical significance per se does not necessarily imply that this approach is also the most valid and optimal. It is to emphasize, however, that we could in principle (internally) replicate our findings here, using two different approaches, i.e. a text-based and a sentence-based, strategy of detecting similarity between dream reports and plans, in combination with a statistical assessment of different target parameters (number of above-criterion similarity reports vs. forced choice allocation of the maximum similarity report). This mutual confirmation further corroborates the validity of our approach, although there may be other more optimal strategies for detecting semantic similarity. An example is BERTScore [50] which is a language generation evaluation metric based on BERT contextual embeddings. In contrast to our approach, in which we compute cosine similarities between embeddings of task plans and dream reports, it computes the similarity on a token level, taking into account their context via contextual embeddings, i.e. a strategy potentially allowing for more fine-grained comparisons between dream reports and task plans. Generally, the use of large language models for analyzing dream content is in its beginnings but, eventually, may turn out a promising tool also for other topics of dream research such as the differentiation of reports of dreams versus more or less emotional wake experiences as well as the differentiation of dream reports among individuals, with potentially important therapeutical implications. Whatever the case, GermanBERT administered to the present data set revealed results confirming our a priori hypotheses. Nevertheless, our approach and findings require further confirmation and external validation, ideally through application to other similar data sets, although other validation strategies are conceivable.

## Supplementary Material

zpae088_suppl_Supplementary_Materials

## Data Availability

Data supporting the findings of this study are available from the corresponding author upon request.

## Notes

This paper is part of the Festschrift in honor of Dr. Robert Stickgold. The open-access publishing fee for this paper was contributed by the Furman University Sleep Laboratory.
