# Supplementary material for: The influence of intentions on dream content
Source: Sleep Adv. 2024 Nov 28;5(1):zpae088. doi: 10.1093/sleepadvances/zpae088 (PMC11697393; doi:10.1093/sleepadvances/zpae088)
Supplement: zpae088_suppl_Supplementary_Materials [file zpae088_suppl_supplementary_materials.docx]

**The Influence of Intentions on Dream Content**

Julia Fechner^1^, Maren Born^1^, Massimiliano Mancini^2^, Zeynep Akata^3,4^, Philipp Haag^1^, Susanne Diekelmann^1,5,†^ and Jan Born^1,6,7,8,†^

^1^ Institute of Medical Psychology and Behavioral Neurobiology, University of Tübingen, Tübingen, Germany

^2^ Multimedia and Human Understanding Group, Department of Information Engineering and Computer Science, University of Trento, Trento, Italy

^3^ Chair of Interpretable and Reliable Machine Learning, Technical University of Munich, Munich, Germany

^4^ Institute for Explainable Machine Learning, Helmholtz Munich, Munich, Germany

^5^ Department of Psychiatry and Psychotherapy, University Hospital Tübingen, 72070, Tübingen, Germany

^6^ Werner Reichert Center for Integrative Neuroscience, University of Tübingen, Tübingen, Germany

^7^ German Center for Mental Health (DZPG), Tübingen, Germany

^8^ German Center for Diabetes Research (DZD), Institute for Diabetes Research & Metabolic Diseases of the Helmholtz Center Munich at the University Tübingen (IDM), Germany

† shared senior authorship

Correspondence to: Jan Born

Email: jan.born@uni-tuebingen.de

**Supplementary Material**


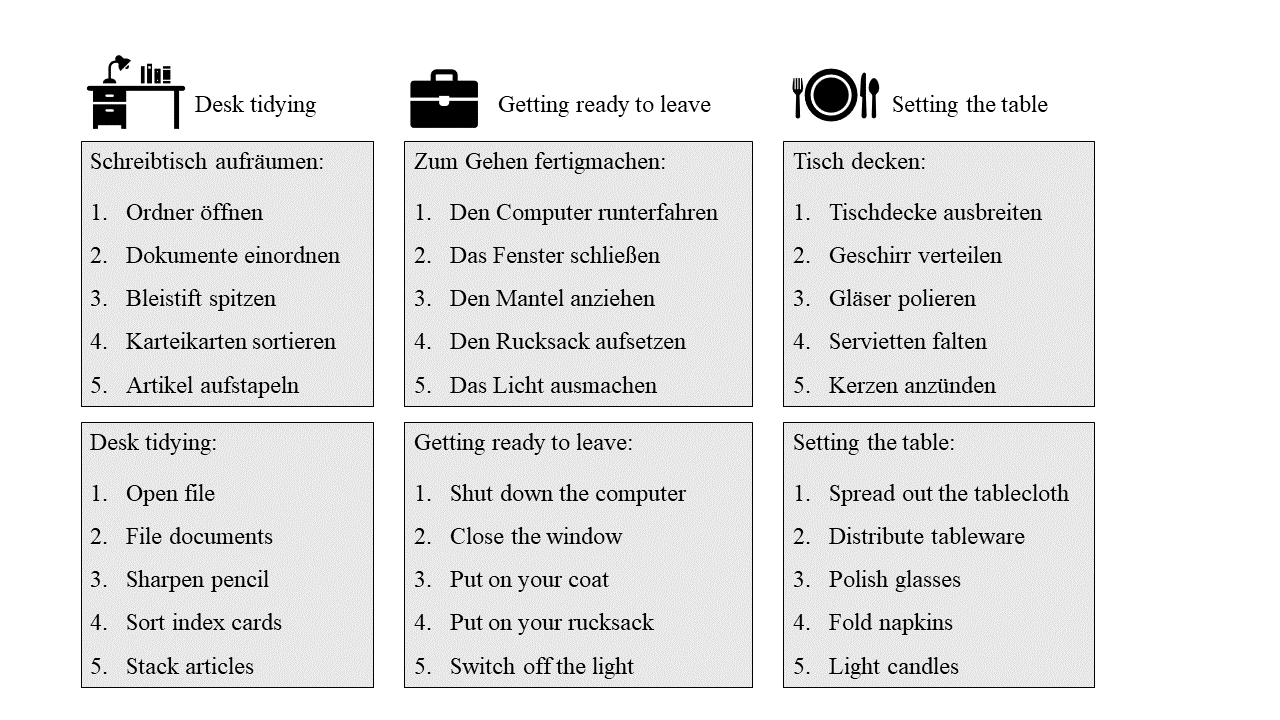


**Supplementary Figure 1: Task plans.** Upper panels: original German version, lower panels: English translation.
